# The protective role of family structure for adolescent development in sub-Saharan Africa

**DOI:** 10.1371/journal.pone.0206197

**Published:** 2018-10-29

**Authors:** Oluwaseyi Dolapo Somefun, Clifford Odimegwu

**Affiliations:** Demography and Population Studies Programme, Schools of Public Health and Social Sciences, Faculties of Health Sciences and Humanities, University of the Witwatersrand, Johannesburg, South Africa; London School of Hygiene and Tropical Medicine, UNITED KINGDOM

## Abstract

Several studies have focused on the risk factors associated with adolescent developmental outcomes, but the literature on the role of protective factors at the family and community level for positive adolescent development is scarce, especially in sub-Saharan Africa (SSA). We hypothesize that ensuring a supportive environment for adolescents may result in delayed sexual debut for adolescents in SSA. The relationship between family structure and positive adolescent sexual behaviour, measured as delay in sexual debut, was examined using the bioecological theory framed by a risk and resilience perspective. We used nationally representative data on female and male adolescents (aged 15–17 years) from 12 countries in SSA. We modelled logistic regressions to test for associations between family structure and delayed sexual debut while controlling for other covariates in SSA. The majority (90%) of the young adults delayed sexual debut, and this delay varied by family structure. After controlling for other covariates, adolescents living with neither parent had lower odds of delaying sexual debut although results were only significant for males. Interaction terms with community socio-economic status showed an interaction between community education and males living with neither parent. Future studies must investigate the gender differentials in the relationship between family structure and delayed sexual debut among adolescents in SSA.

## Introduction

Adolescence has been characterized as a period of both risk and opportunity [1]. This phase gives young people the prospects to cultivate behaviours that form a strong foundation for healthy lifestyles during their life-course. It can also be a phase of experimentation, which has resulted in a number of programmes being tailored to the needs and rights of adolescents globally. Interventions at the global, national and local levels have been directed to articulating and meeting the needs of adolescents living in various settings. The key priorities have been school retention, sexual and reproductive health and increasing the age of marriage for girls [2, 3].

A large body of research has documented the developmental outcomes of adolescents globally and in sub-Saharan Africa, concluding that one of the main threats to young people’s health are the risk behaviours they engage in. Recent data suggest that the leading causes of death among young adults in the region can be attributed to HIV/AIDS [4], clandestine abortions [5] and complications during childbirth [6].

Risky sexual activity is at the centre of the various disease burdens among adolescents. This is because a lack of or inconsistent condom use has been regularly associated with HIV/AIDS and unwanted pregnancies [7–9]. Research has also suggested that an early sexual debut (sex before age 14), significantly increases the risk for unintended pregnancy, sexually transmitted infections (STIs) and future risky sexual behaviour [10–12].

Socio-demographic characteristics have been associated with risky sexual behaviours such as early sexual debut. For example, place of residence measured as urban or rural, household wealth status, gender and religion have been found to be associated with early sexual debut [13–16]. Some contextual factors such as community socio-economic status, socio-cultural norms and school environments have also been found to play a role in the initiation of sex before the age of 14 [17–19].

Although there is available information on prevalence of risky sexual behaviours among adolescents, literature on adolescents engaging in positive sexual behaviours in the same environment as their counterparts who do not is missing. There is a growing interest on the interplay between vulnerability and resilience for protective factors to influence youth behavioural outcomes.

As part of this growing body of research, the social contexts in which adolescents are rooted, including the family [20, 21] and school contexts [22, 23], have been identified as potential protective factors for adolescent development. However, the manner in which adolescents' connections to these contexts shape their behaviours is poorly understood and has not been well documented in sub-Saharan Africa (SSA). This lack of evidence calls for action, as adolescents can be powerful agents of personal change and community development.

Studies that have examined protective factors have concluded that adolescents who exhibit high levels of self-esteem [24] and have high connectedness to educational aspirations [25] are more likely to engage in behaviours that improve their health and development. Religious attendance and beliefs have also been documented to be associated with positive sexual behaviour among adolescents [26, 27]. Some studies have found parental supervision and adolescent involvement and association with peers who display positive attitudes to be protective against early sexual intercourse [28]. In addition, the presence of strict family rules, adolescents’ opinions on parental condemnation of premarital sex and their contentment with rapport with their mothers were significantly related to adolescent sexual abstinence, reduced frequency of sexual activity, and more consistent use of contraceptives [29].

Although risk and protective factors are well-known, the interaction between these two factors is not completely understood. The literature has shown that some adolescents who reside up in single-parent households amid poverty and little monitoring or support do not become involved in risky behaviours [30, 31]. These conclusions imply that some protective factors working concurrently with risk factors may offset and even contradict the most harmful effects of negative factors. Thus, an important but less researched question in the research devoted to adolescent development is the following: which protective factors mitigate the risk of adolescents engaging in sexual debut at an early age?

At the individual level, educational achievement and self-efficacy have been documented to be protective factors for adolescent sexual health [32, 33], and at the family level, family structure (two-parent households, male-headed households) has been consistently associated with positive sexual behaviours [34]. Although a number of studies document a positive relationship between family structure and positive sexual behaviour, other studies find a negative relationship or no association. For instance, Defo and Dimbuene [35] documented the protective effects of two-parent families using longitudinal data in Cameroon while another study in Uganda reported the protective effect of having resident biological fathers for delayed sexual debut among adolescent girls [36].

### Theoretical underpinning

This paper will utilize the protective factor model in the risk and resilience framework. The risk and resilience framework is advantageous, as it recognizes the complexity of adolescents and the environments in which they are nested [37]. The risk and resilience framework subjects the development of adolescents to factors within and outside the adolescent. Resilience refers to the process that allows an individual to avoid the negative effects associated with risk exposure and display positive outcomes [38].

The protective factor model suggests that protective factors may help reduce the effects of risky behaviours by supporting adolescents psychologically, which may include their self-esteem or their perceptions of exposure to risk [39]. The protective factor model proposes that individual strengths or resources available in the environment decrease the deleterious effects challenging young people [40]. This model explains that protective factors operate in a collaborating manner to shield a young person’s exposure to risk. Two ways in which these interact are; risk-protective and protective-protective. Risk-protective models show that protective factors function in ways that decrease the relationship between risks and adverse outcomes. Protective-protective models function as protective agents for a particular outcome. This model can be used by examining an interaction effect between risk and protective factors for predicting sexual behaviour. This has been established by a number of research [41, 42] which have documented the protective effect of family structure in the sexual behaviour outcomes of youth.

Based on this background, the present analysis hypothesized that; 1) adolescents living with both parents would be more likely to delay sexual debut compared to counterparts living with single parent (mother or father) or neither parent; 2) even in the presence of community poverty, adolescents living with both patents would delay sexual debut compared to their counterparts living with single parent (mother or father) or neither parent and 3) community education would strengthen the positive association between living with both parents and delayed sexual debut. The interactions between family structure and community poverty would indicate the risk-protective model while the interactions between family structure and community education indicate the protective-protective model.

We also expect these associations to differ for males. Although gender norms are generally strengthened culturally, the family is the basic unit where adolescents first come close to understanding gender roles [43]. In SSA, young males are usually expected to be sexual risk takers and may more liberal which may influence their sexual behaviours compared to their female counterparts who are expected to be submissive in sexual interactions and ignorant of sexual matters [44]. This may influence the effect family structure would have on male sexual behaviour.

## Materials and methods

### Data source

This paper uses data from the nationally representative Demographic and Health Surveys (DHS), conducted in different countries in SSA. DHS data allows for international comparability of results because the DHS programme employs similar methodologies in data collection across different countries and settings (The DHS Program, 2017). To qualify, a country needed to have had a DHS after 2011; the choice of 2011 as the cut-off year was to provide up-to-date information on the sexual behaviour of adolescents. The countries included are the following: Cameroon 2011, Gabon 2012 and Congo DRC 2013–14, representing Central Africa; Kenya 2014, Rwanda 2014 and Tanzania 2014–15, representing East Africa; Namibia 2013, Zambia 2013–14 and Zimbabwe 2015, representing Southern Africa; and Liberia 2013, Niger 2012 and Nigeria 2013, representing West Africa. Adolescents who had never had sex were excluded from the sample. The present analysis is based on a sample of unmarried females and males aged 15–17 years. The full details of analytic sample have been presented in S1 Appendix.

### Variables of interest

#### Outcome variable

The dependent variable, delayed sexual behaviour, was measured as a binary outcome. Age at first sex was derived from the question “how old were you when you had sex for the first time?” This was a continuous variable. Sexual debut at 14 years or younger was defined as early sexual debut. We are interested in adolescents who delayed sexual debut; therefore, those who delayed sex and who have never had sex were coded as “1”. We have chosen this cut-off because of our study population and also because it has been used in some other studies in SSA [45, 46].

#### Independent variables

The key independent variable is family structure, which was operationalized as adolescents “living with both parents”, “living with mother alone”, “living with father alone” and “living with neither parent”. This variable was measured by using data from adolescents aged 15–17 from the women recode and merging with the household member recode. This was done because parental survival and coresidence questions are only asked about children under 18 which are available in the household member recode.

Other explanatory variables used included place of residence measured as urban and rural, educational status, household wealth status, work status, sex of household head and exposure to mass media. Wealth status, which is a proxy for household socioeconomic status was captured through a wealth index based on household possessions and amenities which were available in the DHS [47]. Mass media messages exposure was measured using questions that asked respondents the; frequency of reading newspapers or magazines; frequency of listening to the radio; and frequency of watching television. Responses ranged from; 0 if ‘not at all’; 1 if ‘less than once a week’; 2 if ‘at least once a week’ and 3 if ‘almost every day’. Response scores ranged from 1 to 3 for each question in the index, resulting in a 6 -point scale. (Cronbach’s alpha = 0.69 and 0.67 for males and females respectively). The scores were then used to create the break points that defined media utilization tertiles as low and high. The community level variables examined included community poverty and community education. These variables were constructed by aggregating the individual and household level variables at the primary sampling units which served as a proxy for community level variables.

### Statistical method

Descriptive statistics for all explanatory variables were cross-tabulated by early and delayed sexual debut. To examine the association between family structure and sexual debut, logistic regression models were fitted in five stages in order to explore the influence of individual- and community-level characteristics on delayed sexual debut. A one-variable model which considered family structure, control variables and the interaction variables were modelled independently in Model 1. Model 2 considered family structure while adjusting for individual level variables while contextual variables were accounted for in Model 3. Model 4 is the full model which accounted for family structure, individual level variables and the contextual factors. Finally, Model 5 was fitted with interaction terms to examine whether the effects of family structure differed by contextual characteristics. Analysis was conducted using Stata software (version 14). To account for under- and oversampling of some settings in the country, weighting factors were applied at various levels of analysis.

#### Ethical consideration

Data from the Demographic and Health Survey (DHS) can be downloaded from the website and is free to use by researchers for further analysis. In order to access the data from the DHS, a written request was submitted to the DHS MACRO and permission was granted to use the data for the survey countries by the DHS program which keeps the archived data. The Demographic and Health Surveys follow similar and standard data collection procedures which ensure data anonymity and guarantees the privacy of all participants. They also ensure that the survey adheres to the U.S. Department of Health and Human Services regulations for the protection of human subjects, whilst the host country ensures that the survey observes local laws and norms. The goals of the survey were explained to the respondents during data collection and written informed consent was obtained from respondents.

## Results

### Descriptive results

The descriptive results in Table 1 show that there was no difference in the delayed sexual debut of males and females (94% for females and 93% for males). By family structure, about 96% of females living with both parents have delayed sexual debut compared to 91% of females living with neither parents who delayed sexual debut. This result was slightly different for males where 94% of males living with both parents delayed sexual debut compared to 92% living with neither parents.

**Table 1:** Descriptive statistics by sexual debut.

	Female (N = 24,779)		Male (N = 15,465)	
Family Structure	Sex before 14 (6%)	Sex after 14 (94%)	Sex before 14 (6%)	Sex after 14 (94%)
Living with both parents	356 (4.0)	8,637 (96.0)	356 (4.0)	8,637 (96.0)
With mother alone	215 (4.8)	4,291 (95.2)	215 (4.8)	4,291 (95.2)
With father alone	66 (5.4)	1,161 (94.6)	66 (5.4)	1,161 (94.6)
With neither parents	765 (9.0)	7,763 (91.0)	765 (9.0)	7,763 (91.0)
**Place of residence**				
Urban	444 (4.5)	9,495 (95.5)	444 (4.5)	9,495 (95.5)
Rural	1,049 (7.1)	13,652 (92.9)	1,049 (7.1)	13,652 (92.9)
**Educational attainment**				
No education	315 (12.6)	2,192 (87.4)	315 (12.6)	2,192 (87.4)
Primary	695 (7.4)	8,670 (92.6)	695 (7.4)	8,670 (92.6)
Secondary & Higher	483 (3.8)	12,277 (96.2)	483 (3.8)	12,277 (96.2)
**Household wealth status**				
Poor	806 (8.7)	8,454 (91.3)	806 (8.7)	8,454 (91.3)
Middle	287 (5.8)	4,687 (94.2)	287 (5.8)	4,687 (94.2)
Rich	400 (3.8)	10,006 (96.2)	400 (3.8)	10,006 (96.2)
**Work status**				
Not working	886 (5.1)	16,380 (94.9)	886 (5.1)	16,380 (94.9)
Working	485 (9.1)	4,813 (90.9)	485 (9.1)	4,813 (90.9)
**Sex household head**				
Male	1,086 (6.3)	16,042 (93.7)	1,086 (6.3)	16,042 (93.7)
Female	407 (5.4)	7,105 (94.6)	407 (5.4)	7,105 (94.6)
**Media Exposure**				
No	1,000 (6.7)	14,029 (93.3)	1,000 (6.7)	14,029 (93.3)
Yes	376 (4.9)	7,269 (95.1)	376 (4.9)	7,269 (95.1)
**Region**				
Central Africa	478 (8.6)	5,099 (91.4)	478 (8.6)	5,099 (91.4)
East Africa	377 (5.2)	6,863 (94.8)	377 (5.2)	6,863 (94.8)
West Africa	468 (6.5)	6,782 (93.5)	468 (6.5)	6,782 (93.5)
Southern Africa	170 (3.7)	4,403 (96.3)	170 (3.7)	4,403 (96.3)

By socio-economic status, 96% of females who had secondary and higher education delayed sexual debut compared to 87% with no education. This result was different for males where 93% of males with secondary and higher education delayed sexual debut compared to 97% with no education. By region, delayed sexual debut ranged from 89% among males in East Africa to 99% among males in West Africa.

### Bivariate association

Model 1 of Table 2 and Table 3 presents the bivariate associations. The results indicate that living with neither parent was a risk factor for delayed sexual debut. Females (OR– 0.86, CI– 0.73–1.00) and males (OR– 0.65, CI– 0.55–0.76) living with neither parent had significantly lower odds of delaying sexual debut but this association was only significant for males. Similarly, for males (OR– 0.70, CI– 0.58–0.83), living with mother alone was significantly associated with lower odds of delayed sexual debut.

**Table 2:** Unadjusted and adjusted odds of the association between family structure and delayed sexual debut among females.

	Model 1	Model 11	Model 111	Model IV	Model V
**Family Structure**					
Living with both parents					
With mother alone	0.90 (0.75–1.07)	1.00 (0.78–1.27)	0.91 (0.76–1.09)	1.01 (0.79–1.30)	0.99 (0.65–1.50)
With father alone	0.83 (0.62–1.11)	0.74 (0.56–1.00)	0.84 (0.62–1.12)	0.76 (0.57–1.03)	0.56 (0.31–0.99)
With neither parents	0.86 (0.73–1.00)	0.85 (0.71–1.03)	0.82 (0.70–0.96) **	0.83 (0.68–1.00)	0.74 (0.52–1.04)
**Place of residence**					
Urban					
Rural	0.72 (0.63–0.82) ***	1.01 (0.85–1.20)		0.95 (0.79–1.14)	0.95 (0.79–1.14)
**Educational attainment**					
No education					
Primary	0.48 (0.34–0.69) ***	0.46 (0.32–0.66) ***		0.51 (0.35–0.76) ***	0.52 (0.35–0.76) ***
Secondary & Higher	0.96 (0.67–1.37)	0.74 (0.50–1.08)		0.87 (0.58–1.29)	0.87 (0.58–1.29)
**Household wealth status**					
Poor					
Middle	1.23 (1.04–1.46) *	1.14 (0.95–1.38)		1.10 (0.91–1.34)	1.10 (0.91–1.33)
Rich	1.78 (1.54–2.06) ***	1.51 (1.24–1.83) ***		1.42 (1.16–1.73) ***	1.42 (1.16–1.74) ***
**Work status**					
Not working					
Working	0.46 (0.43–0.57) ***	0.55 (0.47–0.64) ***		0.58 (0.49–0.68) ***	0.58 (0.49–0.68) ***
**Sex household head**					
Male					
Female	0.89 (0.78–1.01)	0.90 (0.75–1.10)		0.89 (0.73–1.08)	0.89 (0.73–1.08)
**Media Exposure**					
No					
Yes	1.09 (0.95–1.26)	0.99 (0.85–1.15)		0.89 (0.76–1.05)	0.89 (0.76–1.05)
**Region**					
Central Africa					
East Africa	1.25 (1.05–1.48) **		1.26 (1.04–1.50) **	1.56 (1.26–1.94) ***	1.56 (1.26–1.94) ***
West Africa	1.94 (1.59–2.37) ***		2.03 (1.64–2.51) ***	1.92 (1.54–2.38) ***	1.92 (1.54–2.39) ***
Southern Africa	1.85 (1.55–2.21) ***		1.87 (1.55–2.25) ***	1.81 (1.49–2.20) ***	1.81 (1.50–2.20) ***
**Community Poverty**					
Low					
High	0.80 (0.71–0.91) ***		0.86 (0.72–1.03)	0.96 (0.7–1.16)	0.87 (0.67–1.15)
**Community Education**					
Low					
High	1.21 (1.06–1.38) ***		1.09 (0.91–1.30)	0.94 (0.78–1.14)	0.90 (0.69–1.18)
**Interaction effects**					
**Family structure and community poverty**					
With mother alone * high					1.04 (0.69–1.55)
With father alone * high					1.40 (0.74–2.64)
With neither parents * high					1.08 (0.76–1.53)
**Family structure and community education**					
With mother alone * high					1.00 (0.67–1.50)
With father alone * high					1.35 (0.71–2.55)
With neither parents * high					1.16 (0.82–1.65)

* = p<0.1 (significant at 10%).

** = p <0 .05 (significant at 5%).

*** = p<0.01 (significant at 1%).

Na–omitted in Model 5 to prevent multi-collinearity.

**Table 3:** Unadjusted and adjusted odds of the association between family structure and delayed sexual debut among Males.

	Model 1	Model 11	Model 111	Model IV	Model V
**Family Structure**					
Living with both parents					
With mother alone	0.70 (0.58–0.83) ***	0.80 (0.61–1.05)	0.81 (0.68–0.98)	0.86 (0.65–1.13)	0.87 (0.56–1.36)
With father alone	0.90 (0.68–1.18)	0.97 (0.70–1.33)	0.87 (0.65–1.1)	0.87 (0.63–1.22)	0.95 (0.49–1.81)
With neither parents	0.65 (0.55–0.76) ***	0.75 (0.62–0.91) ***	0.74 (0.63–0.88) ***	0.82 (0.67–1.01)	0.65 (0.46–0.93) *
**Place of residence**					
Urban					
Rural	0.91 (0.79–1.04)	0.84 (0.69–1.01)	0.93 (0.76–1.14)	0.93 (0.76–1.14)	0.94 (0.76–1.14)
**Educational attainment**					
No education					
Primary	0.22 (0.13–0.36) ***	0.20 (0.11–0.35) ***	0.50 (0.28–0.91) *	0.50 (0.28–0.91) *	0.50 (0.28–0.91)
Secondary & Higher	0.27 (0.16–0.45) ***	0.27 (0.15–0.48) ***	0.57 (0.31–1.04)	0.57 (0.31–1.04)	0.57 (0.31–1.04)
**Household wealth status**					
Poor					
Middle	1.10 (0.93–1.30)	1.15 (0.94–1.40)	1.12 (0.90–1.38)	1.12 (0.91–1.38)	1.12 (0.91–1.39)
Rich	1.21 (1.05–1.40) ***	1.07 (0.87–1.31)	1.07 (0.86–1.33)	1.07 (0.86–1.33)	1.07 (0.86–1.33)
**Work status**					
Not working					
Working	0.67 (0.59–0.77) ***	0.66 (0.56–0.77) ***	0.64 (0.55–0.76) ***	0.64 (0.55–0.76) ***	0.64 (0.55–0.76) ***
**Sex household head**					
Male					
Female	0.83 (0.72–0.95) **	0.93 (0.74–1.16)	0.97 (0.77–1.22)	0.97 (0.77–1.22)	0.97 (0.77–1.22)
**Media Exposure**					
No					
Yes	0.63 (0.54–0.72) ***	0.64 (0.55–0.74) ***	0.65 (0.54–0.77) ***	0.65 (0.54–0.77) ***	0.64 (0.54–0.77) ***
**Region**					
Central Africa					
East Africa	0.93 (0.79–1.10)		0.96 (0.81–1.15)	1.19 (0.90–1.57)	1.19 (0.90–1.56)
West Africa	8.60 (6.27–11.77) ***		9.12 (6.50–12.79) ***	8.55 (5.78–12.61) ***	8.52 (5.78–12.56) ***
Southern Africa	1.32 (1.12–1.56) ***		1.31 (1.10–1.56) ***	1.24 (0.96–1.59)	1.23 (0.96–1.58)
**Community Poverty**					
Low					
High	0.90 (0.79–1.03)		1.00 (0.85–1.17)	1.01 (0.84–1.22)	1.02 (0.77–1.34)
**Community Education**					
Low					
High	1.16 (1.02–1.32) *		1.10 (0.94–1.30)	1.18 (0.98–1.42)	1.00 (0.76–1.32)
**Interaction effects**					
**Family structure and community poverty**					
With mother alone * high					0.89 (0.58–1.38)
With father alone * high					0.82 (0.41–1.65)
With neither parents * high					1.13 (0.76–1.6)
**Family structure and community education**					
With mother alone * high					1.09 (0.71–1.69)
With father alone * high					1.04 (0.52–2.10)
With neither parents * high					1.46 (0.84–2.17) ***

* = p<0.1 (significant at 10%).

** = p <0 .05 (significant at 5%).

*** = p<0.01 (significant at 1%).

By region, female adolescents from East (OR– 1.25, CI– 1.05–1.48), West (OR– 1.94, CI– 1.59–2.37) and Southern (OR– 1.85, CI– 1.55–2.21) Africa had higher odds of delaying sexual debut compared to their counterparts in Central Africa. These results were similar for males in West (OR– 8.60, CI– 6.27–11.77) and Southern Africa (OR– 1.32, CI– 1.12–1.56) who had significantly higher odds of delayed sexual debut. However, the results differed for males in East Africa (OR– 0.93, CI– 0.79–1.10) who had lower odds of delaying sexual debut.

At the bivariate level, place of residence, educational attainment, work status, wealth status sex of household head and media exposure were also associated with delayed sexual debut.

The contextual variables showed similar results for both males and females. Community poverty was associated with lower odds of delayed sexual debut while community education showed a positive association with delayed sexual debut.

### Multivariate association

The results in Model II of Table 2 and Table 3 show the adjusted odd ratios of family structure and individual covariates. In this model, the association between family structure and delayed sexual debut was negative but remained insignificant for females. However, males living with neither parent (OR– 0.75, CI– 0.62–0.91) had reduced odds of delaying sexual debut.

Other individual covariates associated with delayed sexual debut were socio-economic status, sex of household head and media exposure which differed by gender. For instance, females (OR– 1.51, CI– 1.24–1.83) living in households that belonged to the rich quintile had significantly higher odds of delaying sexual debut but this association was not significant for males (OR– 1.07, CI– 0.87–1.31). There was no association between media exposure and delayed sexual debut among females but males (OR– 0.64, CI– 0.55–0.74) who reported being exposed to mass media had lower odds of delaying sexual debut.

Controlling for contextual characteristics alone in Model 3 of Tables 2 and 3, the relationship between family structure and delayed sexual among females becomes significant for females. Females living with neither parent (OR– 0.82, CI– 0.70–0.96) had reduced odds of delaying sexual debut. This association was similar for males (OR– 0.74, CI– 0.63–0.88).

The significant association between family structure and delayed sexual debut changed in the adjusted model (model 4) of Tables 2 and 3. The full model also showed some other characteristics that were associated with delayed sexual debut for males and females. Primary education showed a negative relationship with delayed sexual debut for females (OR– 0.51, CI– 0.35–0.76) and males (OR– 0.50, CI– 0.28–0.91). Females in East, West and Southern Africa had significantly higher odds of delaying sexual debut compared to their counterparts in Central Africa. This association was similar for males but only significant for males in West Africa. The relationship between family structure and delayed sexual debut remained unchanged in model 5 which had the interaction term.

### Analysis of interaction effects

The result in Table 4 shows that the odds ratio of the association between family structure and delayed sexual debut was 0.65 comparing ‘living with neither parent’ with ‘living with both parents’ among those with low community education and 0.95 among those with high education, a statistically significant interaction.

**Table 4:** Interaction terms between family structure and community education.

Main effects (Family structure)	Low education	Community education (High)
**living with mother alone**	0.87	1.09
**living with father alone**	0.95	1.04
**living with neither parent**	0.65	1.46

## Discussion

Scholars exploring adolescents’ development have argued that the social structures in which adolescents exist in have important consequences in terms of their sexual behaviour [48, 49]. As a key agent of socialization, the family has been documented to exert a strong and multifaceted influence on adolescents; albeit with numerous underlying mechanisms. As stated in the introduction, the objective of this paper was to identify the protective effect of living in a two parent household on delayed sexual debut; whether living in a two parent household would still be positively associated with delayed sexual debut in the face of community poverty and whether community education would strengthen the positive association between living with two parent and delayed sexual debut. The paper also sought to examine whether these associations differed by gender. Our results are in line with the protective model of the risk and resilience framework.

This paper provides evidence of the importance of family and community determinants of sexual behaviour among female and male adolescents. About one third of the adolescents resided with both parent and majority (90%) of the adolescents delayed sexual activity. Our findings are in line with hypothesis. We found living with both parents to be a protective factor for delayed sexual debut among adolescents and results remained unchanged even in the presence of community poverty and education. These associations differed by gender.

### Family protective factors

Our findings on the influence of family structure on adolescent sexual behaviour are similar to what some other studies have documented in developed [50] and developing countries [36, 51, 52]. Adolescents living with neither parent were less likely to delay sexual debut compared to their counterparts living with both parents. Similar results were seen for adolescents living with mother or father alone. Reasons for the protective effect of living with both parents have been established in the literature. The presence of two parents in the household may increase the earnings in the household thereby reducing the odds of female adolescents getting involved in risky behaviours such as sexual experimentation at an early age. Having two parents in the household may also allow for division of labour where either the mother or father has enough time to communicate with adolescent which may influence self-esteem. The positive association between self-esteem and positive sexual behaviours have been well established. Similar to other studies [30], the influence of family structure on sexual debut of adolescents was stronger for females than it was for males. This means that factors that protect females are different from factors that protect males. One possible explanation is that women are required to be conservative regarding their sexual behaviour while promiscuity may be tolerated and encouraged among men in some African settings. We also suggest that females may be closer with family members compared to males which may influence their sexual decisions.

### Community factors

The role of community variables for adolescent development outcomes have been mixed. This was highlighted in our results. In the presence of community poverty, living with neither parent reduced the odds of delayed sexual debut for females. This is not surprising as community poverty may be associated with the type of information available to the community. Poorer communities may have decreased access to media campaigns that promote abstinence or positive sexual behaviours. Poverty also leads to scarcity of resources and opportunity structures available in communities that may help improve the livelihoods of basic institutions in the communities [53]. Another mechanism through which community poverty may influence sexual behaviour of adolescent delayed sexual behaviour is through the transfer of social norms and behaviours. Poverty has been associated with increased odds of risky sexual behaviour and members that share the same local environment may perceive risky behaviours as the norm which may influence the behaviours of adolescents living in the community. We had expected that community education would result in delayed sexual debut among adolescents but our results showed that females living with both parents and residing in communities with a high percentage of educated women had lower odds of delaying sexual debut. This result is surprising as we expected that community education would strengthen the association between adolescents living with both parents and delayed sexual debut. The results for males showed that community education was a protective factor for delayed sexual debut although it was not significant. Although the period of adolescence is associated with independence, interaction with peers and direct access to the environment, the effects of community characteristics may matter more during childhood than during adolescence which may explain our unexpected results [54].

The interaction terms were not significant for females but there was an interaction between family structure and community education for males. The observed moderately strong lower odds of delayed sexual debut for those living with neither parent among those with lower community education is almost absent among participants in communities with high education. This implies that interventions focusing on the role of family structure on delayed sexual debut may focus more on those adolescents not living with both parents in communities with lower education.

At the individual level, secondary and higher education was a risk factor for delayed sexual debut among adolescents which contradicts what is in the literature [33, 55]. We suggest that this could be due to the fact that higher educational attainment may increase exposure of adolescents which may have a negative effect on sexual behaviour. This exposure could be access to mobile phones or internet. This suggestion has been partly explained by the negative association seen between work status and delayed sexual debut. Males and females who were working had lower odds of delayed sexual debut. Adolescents who are working may be more exposed to older adults and also peers which may influence decision to delay sexual debut.

Region was significantly associated with delayed sexual for female and male adolescents. Males in Southern Africa were more likely to delay sexual debut compared to their counterparts in Central Africa. This could be based on the fact that notable policies have been implemented towards promoting responsible sexual behaviour and practice among young people in Southern Africa. For example, Namibia has increased its spending on HIV prevention strategies and educates young adults in Schools [56]. Zambia is another country in Southern Africa which has invested a lot in mass media to improve sexual behaviours of youth and studies have documented the improved health outcomes [57].

## Limitations

We are aware that this study may have limitations. First, due to the cross-sectional nature of the data, the analysis was only able to determine correlation and association and thus prevents any conclusions from being drawn about causation between family-level factors and adolescent SRH outcomes. The effect of family structure on adolescent sexual behaviour is complex and cannot be understood by isolating and focusing on a single construct such as living arrangement, but rather by examining a broad set of factors that impact adolescent sexual behaviour. Another limitation is our measure of community factors, which focuses on the economic dimension and wealth status; this measure has been used as a proxy and may differ in the regions studied.

## Conclusion

The evidence from this study implies that more than 90% of adolescents in SSA are delaying age of sexual debut. Multivariable analyses showed that even after adjusting for community factors, living with both parent was a protective factor for delayed sexual debut among females but not for males. The gender differential in the relationship between family structure and adolescent development in SSA is a fundamental point for future research. Future work on adolescent sexual and reproductive health can potentially explain why the protective effect educational attainment on delayed sexual debut differs by gender. This multifaceted association between family structure, gender and adolescent sexual behaviour permits additional investigation, as it has important policy and programmatic implications for sub-Saharan Africa, which is experiencing changes in family structure dynamics and an increasing number of young people. The paper concludes that existing programs and policies must be sustained for all adolescents to maintain the progress being made in positive adolescent development. Finally, this paper presents new evidence on the relationship between family structure, contextual characteristics and delayed sexual debut among adolescents in SSA.

## Supporting information

S1 Appendix Sample size by country. (DOCX)
