# Correction: The protective role of family structure for adolescent development in sub-Saharan Africa

**DOI:** 10.1371/journal.pone.0221723

**Published:** 2019-08-22

**Authors:** Oluwaseyi Dolapo Somefun, Clifford Odimegwu

In Table 1, the same values are shown in the Female and Male column. Please see the correct Table 1 here.

**Table 1:** Descriptive statistics by sexual debut.

	Female		Male	
Family Structure	Sex before 14	Sex after 14	Sex before 14	Sex after 14
Living with both parents	356 (4.0)	8,637 (96.0)	396 (6.3)	5,927 (93.7)
With mother alone	215 (4.8)	4,291 (95.2)	205 (7.7)	2,467 (92.3)
With father alone	66 (5.4)	1,161 (94.6)	68 (6.3)	1,010 (93.7)
With neither parents	765 (9.0)	7,763 (91.0)	325 (8.4)	3,541 (91.6)
**Place of residence**				
Urban	444 (4.5)	9,495 (95.5)	378 (6.5)	5,465 (93.5)
Rural	1,049 (7.1)	13,652 (92.9)	717 (7.5)	8,883 (92.5)
**Educational attainment**				
No education	315 (12.6)	2,192 (87.4)	25 (2.6)	952 (97.4)
Primary	695 (7.4)	8,670 (92.6)	504 (8.3)	5,535 (91.7)
Secondary & Higher	483 (3.8)	12,277 (96.2)	566 (6.7)	7,856 (93.2)
Household wealth status				
Poor	806 (8.7)	8,454 (91.3)	409 (7.1)	5,317 (92.9)
Middle	287 (5.8)	4,687 (94.2)	259 (7.5)	3,200 (92.5)
Rich	400 (3.8)	10,006 (96.2)	427 (6.8)	5,831 (93.2)
**Work status**				
Not working	886 (5.1)	16,380 (94.9)	589 (5.8)	9,618 (94.2)
Working	485 (9.1)	4,813 (90.9)	505 (9.8)	4,669 (90.2)
**Sex household head**				
Male	1,086 (6.3)	16,042 (93.7)	762 (6.8)	10,504 (93.2)
Female	407 (5.4)	7,105 (94.6)	333 (8.0)	3,844 (92.0)
**Media Exposure**				
No	1,000 (6.7)	14,029 (93.3)	540 (6.1)	8,273 (93.9)
Yes	376 (4.9)	7,269 (95.1)	419 (9.0)	4,256 (91.0)
**Region**				
Central Africa	478 (8.6)	5,099 (91.4)	380 (7.6)	4,720 (92.6)
East Africa	377 (5.2)	6,863 (94.8)	352 (11.3)	2,773 (88.7)
West Africa	468 (6.5)	6,782 (93.5)	46 (1.4)	3,327 (98.6)
Southern Africa	170 (3.7)	4,403 (96.3)	317 (8.2)	3,528 (91.8)

## References

[1] Somefun OD, Odimegwu C (2018) The protective role of family structure for adolescent development in sub-Saharan Africa. PLoS ONE 13(10): e0206197 10.1371/journal.pone.0206197 30372474 PMC6205637

