# Supplementary material for: The protective role of family structure for adolescent development in sub-Saharan Africa
Source: PLoS One. 2018 Oct 29;13(10):e0206197. doi: 10.1371/journal.pone.0206197 (PMC6205637; doi:10.1371/journal.pone.0206197)
Supplement: S1 Appendix — (DOCX) [file pone.0206197.s001.docx]

**Appendix 1: Sample Size by Country**

|  |  | **Males** | **Females** |
| --- | --- | --- | --- |
| **Country** | **Survey Year** | **Frequency** | **Frequency** |
| Cameroon | 2011 | 1,962 | 2,142 |
| Congo DRC | 2013/14 | 2,204 | 2,376 |
| Gabon | 2012 | 944 | 1,121 |
| Kenya | 2014 | 1,745 | 3.769 |
| Rwanda | 2014/15 | 803 | 1,743 |
| Tanzania | 2014/15 | 579 | 1,749 |
| Namibia | 2013 | 553 | 1,072 |
| Zambia | 2013/14 | 1,994 | 2,168 |
| Zimbabwe | 2015 | 1,302 | 1,348 |
| Liberia | 2013 | 548 | 1,192 |
| Nigeria | 2013 | 2,369 | 4,944 |
| Niger | 2012 | 462 | 1,155 |
| **Total** |  | **15,465** | **24,779** |
